# Cefepime pharmacokinetics in critically ill children with multiple organ dysfunction syndrome using volumetric absorptive microsampling

**DOI:** 10.1128/aac.01736-25

**Published:** 2026-06-04

**Authors:** Victor Amajor, Amanda Bwint, Steven L. Shein, Janet Hume, Courtney M. Rowan, LeeAnne P. Flygt, Matthew P. Malone, Erin K. Stenson, Scott L. Weiss, Matt S. Zinter, Sujatha Kannan, Mary K. Dahmer, Ryan A. Nofziger, Manjunath P. Pai, Athena F. Zuppa, Nathaniel J. Rhodes, Mark W. Hall, Marc H. Scheetz, Kevin J. Downes, on behalf of Pediatric Acute Lung Injury and Sepsis Investigators (PALISI) Network

**Affiliations:** 1 Division of Infectious Diseases, Children’s Hospital of Philadelphia, Philadelphia, Pennsylvania, USA; 2 Division of Critical Care Medicine, Rainbow Babies and Children’s Hospital, Cleveland, Ohio, USA; 3 Division of Critical Care Medicine, University of Minnesota Masonic Children’s Hospital, Minneapolis, Minnesota, USA; 4 Division of Critical Care Medicine, Indiana University School of Medicine at Riley Children’s Health, Indianapolis, Indiana, USA; 5 Division of Critical Care Medicine, University of Iowa Stead Family Children’s Hospital, Iowa City, Iowa, USA; 6 Division of Critical Care Medicine, University of Arkansas for Medical Sciences, Arkansas Children’s Hospital 12215, Little Rock, Arkansas, USA; 7 Division of Critical Care Medicine, Children’s Hospital Colorado, University of Colorado School of Medicine 12225, Aurora, Colorado, USA; 8 Division of Critical Care Medicine, Department of Pediatrics, Nemours Children's Hospital 25401 , Wilmington, Delaware, USA; 9 Department of Pediatrics and Pathology & Genomic Medicine, Sidney Kimmel Medical Center at Thomas Jefferson University, Philadelphia, Pennsylvania, USA; 10 Division of Critical Care Medicine, University of California-San Francisco Benioff Children’s Hospitals, San Francisco, California, USA; 11 Division of Pediatric Critical Care Medicine, Department of Anesthesiology and Critical Care Medicine, Johns Hopkins University School of Medicine 1500 , Baltimore, Maryland, USA; 12 Division of Pediatric Critical Care Medicine, University of Michigan C.S. Mott Children’s Hospital, Ann Arbor, Michigan, USA; 13 Division of Critical Care Medicine, Akron Children’s Hospital, Akron, Ohio, USA; 14 PK Core Laboratory, College of Pharmacy, University of Michigan 15514, Ann Arbor, Michigan, USA; 15 Department of Pediatrics, Perelman School of Medicine of the University of Pennsylvania, Philadelphia, Pennsylvania, USA; 16 Pharmacometric Center of Excellence, Midwestern University 69281, Downers Grove, Illinois, USA; 17 Division of Critical Care Medicine, Nationwide Children’s Hospital, Columbus, Ohio, USA; University of Houston, Houston, Texas, USA

**Keywords:** antibiotics, sepsis, pharmacokinetics

## Abstract

Up to half of critically ill children experience multiple organ dysfunction syndrome (MODS), which worsens outcomes and increases mortality risk. Cefepime is a broad-spectrum antibiotic commonly used in pediatric sepsis, but its pharmacokinetics during MODS has not been evaluated. We performed a prospective, observational study of critically ill children with MODS who were administered cefepime and collected up to 15 whole blood samples over 3 days using volumetric absorptive microsampling (VAMS). We performed nonlinear mixed-effects modeling to develop a population PK model for cefepime in children with MODS. We then reran our final population PK model using estimated plasma concentrations based on a 0.58:1 VAMS:plasma ratio derived *ex vivo*. A two-compartment model with allometric scaling best described the data. Estimated glomerular filtration rate and age were significant covariates on total body clearance and central volume, respectively. Final model parameter estimates for clearance and central volume in VAMS were 5.14 L/h and 20.5 L, respectively, for a 36-kg child. Estimates for clearance and central volume in plasma were 2.97 L/h and 11.51 L, respectively. We provide the first population PK model of cefepime in critically ill children with MODS based on VAMS, along with estimated plasma PK derived from VAMS data. Our study demonstrates the feasibility of using a novel microsampling approach to evaluate antimicrobial PK in critically ill children and provides the groundwork to better understand how this approach can optimize treatment and monitoring in this population.

## INTRODUCTION

Multiple organ dysfunction syndrome (MODS) is a condition characterized by the simultaneous failure or dysfunction of two or more organ systems. Estimates of its prevalence in critically ill pediatric patients vary due to differences in diagnostic criteria, but some studies report that more than half of children in the intensive care unit experience MODS (1). While inciting factors vary, sepsis is its leading cause. Infections, either as the inciting etiology or as a complication of MODS, have been shown to worsen patient outcomes and increase mortality risk (2). Timely and effective dosing of antibiotics is crucial to improving outcomes in this population.

Cefepime is a fourth-generation cephalosporin antibiotic that plays a key role in the treatment of severe bacterial infections (3) and is one of the most common antibiotics prescribed as empiric treatment of suspected sepsis in children. As a β-lactam antibiotic, cefepime’s efficacy depends on the amount of time the unbound drug concentration remains above the minimum inhibitory concentration (MIC) of the infecting pathogen (4). Consensus recommendations and experts advocate for target attainment of 100% time above MIC, especially in critically ill patients (5). However, target attainment in this population can be a challenge, as critical illness often alters the pharmacokinetics (PK) of drugs (6, 7). When the patient’s condition is complicated by complex organ failures, drastic changes in PK parameters, such as volume of distribution and/or clearance, may occur.

MODS poses a challenge to cefepime dosing in critically ill children because of the dramatic impact it can have on drug distribution and elimination. Those with MODS are often the most critically ill, and target attainment is imperative for optimizing outcomes in those with serious bacterial infections. Target attainment is often best ensured via therapeutic drug monitoring (TDM). However, cefepime TDM has not been traditionally available at most institutions in the United States (8). Thus, most dosing is guided predominantly by patient characteristics, such as body weight, but is not typically tailored to important clinical covariates for patients in MODS (e.g., severity of illness). Furthermore, TDM of β-lactam agents in children is challenged by a lack of available clinical assays, difficulties with timing of blood sampling, and limited blood volumes available in pediatric patients. Volumetric absorptive microsampling (VAMS), which involves collection of very small volume (10–20 μL) of dried blood samples, is a potential modality to facilitate transfer of blood samples for TDM at sites that do not have assay capabilities locally (9, 10). Our study aimed to develop a population PK model of cefepime in children with MODS using VAMS, evaluate drug target attainment in this population, and identify patient factors associated with target attainment.

## MATERIALS AND METHODS

### Patients and setting

The PediAtric ReseArch of Drugs, Immunoparalysis, and Genetics during MODS (PARADIGM) study (R01HD095976, PI: Hall) is a multicenter, prospective, observational study of immune function and outcomes in children with MODS conducted across the Pediatric Acute Lung Injury and Sepsis Investigators (PALISI) network. To be eligible for this study, children with acute MODS onset within the past 3 calendar days were enrolled (complete eligibility criteria are shown in Table S1). A subset of PARADIGM patients was enrolled in the Assessment of MODS and Personalized Exposures of Antibiotics (AMPLE) study (R01HD103755, MPI: Downes, Scheetz), an ancillary observational cohort study focused on understanding antibiotic PK and exposures in critically ill children with MODS. Enrollment in AMPLE began in June 2022 at 14 PARADIGM sites. Children enrolled in PARADIGM who received ≥1 of 6 intravenous (IV) antibiotics for standard of care were eligible for participation in AMPLE.

Although enrollment in AMPLE is ongoing, this report is an *a priori* planned population PK model for cefepime after enrollment of ≥30 treated children. For the current analysis, PARADIGM/AMPLE subjects with complete data available who were administered IV cefepime, had ≥1 PK sample collected, and who did not receive continuous renal replacement therapy (CRRT) or plasmapheresis were included; patients who were on extracorporeal membrane oxygenation (ECMO) were included so long as they did not receive concomitant CRRT.

### Cefepime sampling

**Fig 1:**
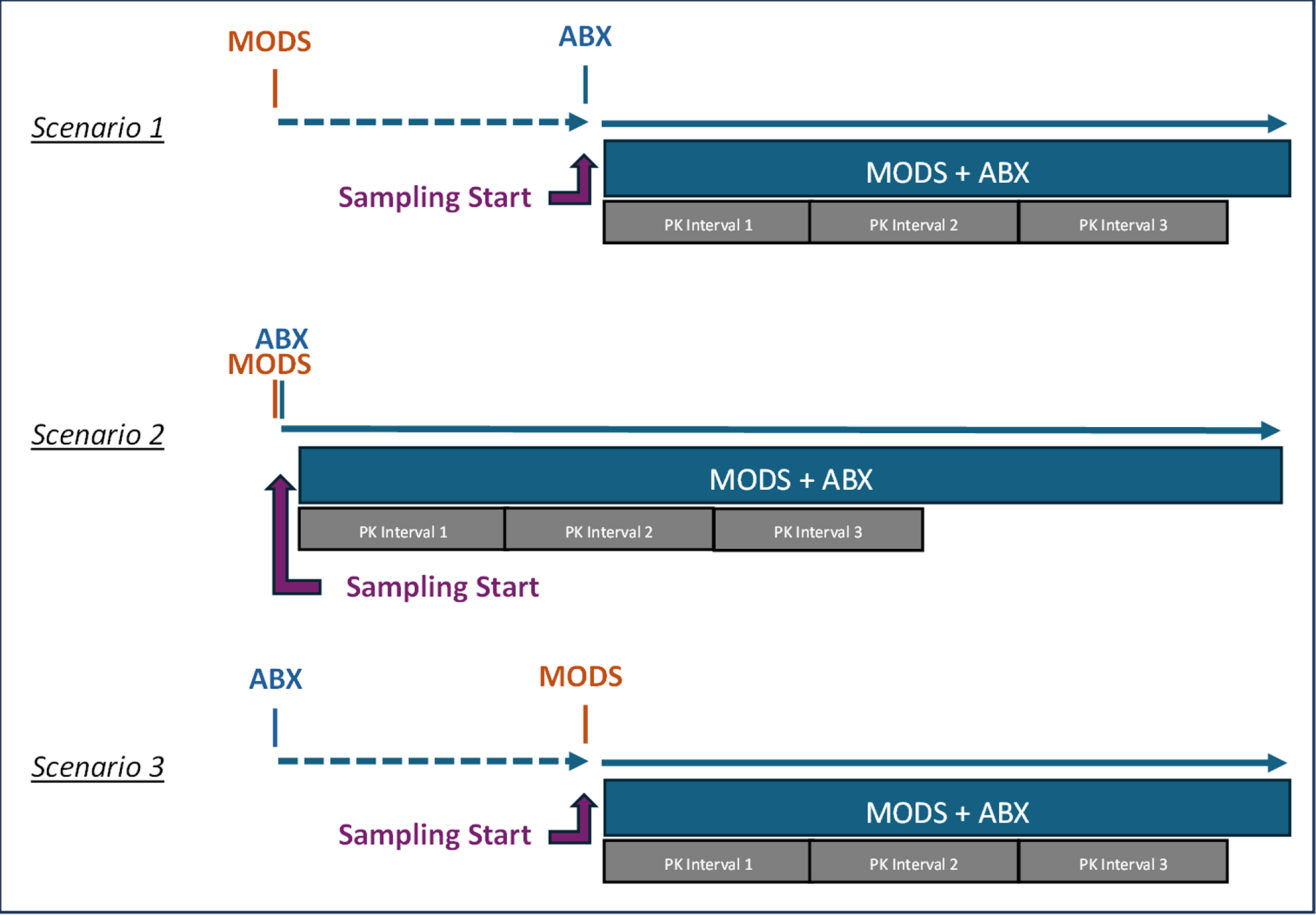
Schematic of pharmacokinetic (PK) sampling in relation to multiorgan dysfunction syndrome (MODS) onset and antibiotic start (ABX). Although antibiotics could start at any time in relation to MODS onset, enrolled patients were eligible for PK sampling only if they were administered an antibiotic after MODS onset (MODS + ABX). All PK sampling began within 72 h of MODS + ABX.

PK samples were collected at up to 15 time points over 3 consecutive calendar days based upon the timing of antibiotic administration during MODS (Fig. 1); since this was an observational study, antibiotic administration could start before, at, or after MODS onset. Whole blood microsamples were obtained (20 μL/sample ×2, 40 μL total) using VAMS devices (Mitra by Trajan Scientific and Medical, Victoria, Australia). After collection, samples were allowed to dry at room temperature for 1–3 h or in a 4°C refrigerator for up to 72 h before being transferred to a −80°C freezer. Our sampling strategy was based on a methodologic validation to support the protocol (10). In brief, total, whole blood cefepime concentrations were measured using a tandem high-performance liquid chromatographic method with mass spectrometry (10); the lower limit of quantification (LLOQ) was 0.1 mg/L. Samples that were collected while cefepime was infusing were deemed to be biologically implausible (e.g., low concentration immediately after an infusion) or were not collected according to the manufacturer’s specifications (e.g., over- or under-filled VAMS tips, improper storage) were excluded from analysis.

### Data collection

MODS Day 0 was defined as the calendar day of MODS onset. Subjects’ Pediatric Risk of Mortality (PRISM III) (11) score at ICU admission was recorded, but did not necessarily coincide with MODS onset. From the electronic medical record, the following data were collected daily for each subject, as available: serum creatinine, cystatin C, hematocrit, fluid status (neutral, positive, or negative volume), as well as use of vasopressors, mechanical ventilation, ECMO, dialysis, and/or plasmapheresis. Organ function scores (Pediatric Logistic Organ Dysfunction [PELOD-2]) (12) and Proulx (13) were calculated daily starting on MODS Day 0 using pertinent data for each scoring system. Based on the highest daily creatinine value available, estimated glomerular filtration rate (eGFR) was calculated for each study day using the Chronic Kidney Disease in Children Under 25 (U25) formula (14). Due to the inconsistent availability of cystatin C across study sites, we calculated the U25 eGFR using only serum creatinine for all subjects.

As part of the PARADIGM study, whole blood *ex vivo* TNF-α response to lipopolysaccharide stimulation, an assessment of immune function (15), was performed on patient blood samples, with lower TNF-α responses indicating a greater degree of innate immune suppression. A TNF-α response <200 pg/mL was classified as immunoparalysis, which represents a severe degree of innate immune suppression (16). Samples were collected multiple times per subject during the study: within three calendar days of MODS onset, 72 h later, and then twice weekly through resolution of organ dysfunction, ICU discharge, or death, whichever occurred first.

### Population PK model development

Nonlinear mixed-effects population PK modeling was conducted using Monolix 2024R1 (Lixoft, Antony, France) to describe cefepime PK in whole blood using VAMS. One and two-compartment structural models with first-order elimination were tested. Between-subject variability (BSV) was evaluated on all clearance and volume parameters, and residual variability was assessed with proportional and combined error structures. Cefepime concentrations that were below the quantification limit (BQL) were interval censored consistent with the M4 method reported by Beal (17, 18).

Model development followed a stepwise approach. We first evaluated compartment and error structures. We then assessed the impact of allometric scaling on model fit. Body weight was evaluated as an allometric scaling factor with powers of 0.75 and 1 for clearance and volume terms, respectively. Finally, we tested the influence of covariates on model parameters. Patient-level data were tested sequentially as covariates on central volume of distribution (V1) and clearance (CL). As appropriate, time-varying covariates (e.g., eGFR, laboratory data, daily organ function scores) were handled as regressors to capture dynamic change in the Monolix workflow. Interpolation was used to account for changes between measured continuous covariate values (e.g., laboratory data) over time. Because organ function scores were calculated daily for PARADIGM, values were applied for each calendar day and were not imputed. When baseline values were not available, the first observation was carried backwards. In cases where patient height was missing (*n* = 2), the CDC reported 50th percentile height for the subject’s age and sex was used (19). Since daily organ function scores (PELOD and/or Proulx scores) were not available prior to MODS onset (i.e., enrollment), we used values that were half of the value on MODS Day 0 for all preceding days.

Covariates were added to the model through a stepwise forward addition method. Additions were considered statistically significant if their inclusion caused a decrease in the −2 *log-likelihood (objective function value [OFV]) of 3.84 units or greater (α = 0.05, df =1). The covariate with the largest significant reduction in OFV was added to the base model and carried forward into further testing. Covariates were sequentially added to the model, so long as they met the reduction of ≥3.84 units in OFV criterion. Once all covariates were added, a sequential backwards elimination process was then conducted to verify covariate significance. If removal of a covariate causes an increase in the OFV of 6.635 units or greater (α = 0.01, df = 1), the covariate was considered significant and was retained in the model. In addition to the OFV criterion, covariates were retained based on reductions in the Corrected Bayesian Information Criteria (BICc), improved precision of parameter estimates, reduced interindividual variability, and visual improvements in goodness-of-fit plots. Together, these criteria supported selection of the most parsimonious, physiologically relevant, and robust population model.

Finally, since VAMS collects whole blood, we re-ran the final population PK model using concentrations adjusted for a whole blood:plasma ratio of 0.58, which was derived based on a series of *ex vivo* experiments (Table S2). This ratio provides estimated plasma PK based on samples collected using VAMS, since plasma is the gold standard biosample for most PK studies and allows for more direct comparisons to published literature.

### Simulation and assessing target attainment in individuals

We used the final VAMS PK model parameters, along with individual subject Bayesian posteriors and dosing data, to estimate individual cefepime exposures via Simulx 2024R1 (Lixoft, Antony, France). For each subject who started cefepime on/after MODS onset, total whole blood cefepime concentrations were simulated for the first 24 h after dosing. This time frame was chosen since daily data collection for PARADIGM started at MODS onset. From these simulated concentrations, the free drug concentrations were extrapolated assuming 20% protein binding (4). We then determined the number/proportion of individuals that maintained free concentrations above MICs (*f*T > MIC) of 2, 4, 8, and 16 mg/L (100% *f*T > MIC) from 0 to 24 h after the start of cefepime.

## RESULTS

### Study population

Forty-five subjects received cefepime, had ≥1 PK sample collected, and had complete demographic and covariate data available. Of these, seven were on renal replacement therapy at the time of PK sample collection and were excluded. One subject was also excluded due to biologically implausible drug concentrations, suggesting collection or storage errors. As a result, 37 subjects were included in the final study population. The median age of this group was 10 years (range: 1 month–17 years) and median weight was 36 kg (range: 4 kg to 213 kg; Table 1); daily characteristics of our study population in relation to PK sampling are provided in Table S3. Two subjects (9-year-old and 13-year-old males) did not have height available, so median values were utilized.

**TABLE 1:** Characteristics of the study population

Variable	Median (range)
Number of subjects, n	37
Number of samples, n	377
Female sex, n (%)	20 (54%)
Age	10y (1m–17y)^*a*^
Weight (kg)	36 (4.3–213.6)
Height (cm)	135 (56–198.1)
Severity of Illness scores	
PELOD-2^*b*^	6 (0–21)
PRISM	11 (0–30)
Proulx^*b*^	2 (0–5)
ECMO treatment,^*a*^ n (%)	4 (11%)
Immunoparalysis (TNF-α response<200 pg/mL) at start of dosing, n (%)	22 (59%)
Albumin (g/dL)	3 (1.6–3.9)
Serum creatinine (mg/dL)	0.48 (0.15–3.08)
U25 GFR (mL/min/1.73 m^2^)^*b*^	87.37 (19.35–278.29)
Dose (mg/kg)	47.6 (9.4–54.3)
Time on drug before sampling (h)	24 (1–199)
MODS Day^*b*^	2 (0–9)

^*a*^ Three subjects were less than 1 year of age (1, 5 and 5 months of age); all were born at 37+ weeks gestation and were >40 weeks at time of study participation.

^*b*^ At the start of sampling.

Among participants, 426 PK samples were collected: 32 samples were excluded from the analysis because they were drawn during drug infusion, 13 were excluded due to sampling and collection errors, and 4 were excluded for being biologically implausible. Thus, 377 samples were included in the final analysis (median 11 samples per subject, range 2–15), and none of these cefepime measurements were BQL. The majority of PK sampling (78%) occurred on MODS Days 0–3; 35 of 37 (95%) of subjects had MODS when PK samples were obtained, while the other two had single organ dysfunction. Figure S1 shows a plot of measured concentrations versus time after dose.

### PK model

A two-compartment model with first-order elimination, allometric scaling (centered on a weight of 36 kg), and a proportional error structure best described the data. BSV on the peripheral volume of distribution (V2) and inter-compartmental clearance (Q) was poorly estimated, so it was fixed to zero for these two parameters. During covariate testing, eGFR on CL and age on V1 significantly improved model fit. Both covariates met forward inclusion and backward elimination criteria and were retained in the final model. Proulx, PELOD, and fluid status met statistical criteria for inclusion as covariates in the model (based on changes in OFV and BICc) but were ultimately not included. PELOD on CL and Proulx on V1 failed to improve BSV on their respective parameters. PELOD on V1 reduced BSV, but its effect size was small and the addition worsened goodness of fit plots. Fluid status on CL and V1 reduced BSV on both parameters, but inclusion led to worse precision of other parameters, suggesting collinearity or overfitting, and so this covariate was not included in the model. See Table S4 for a full summary of the model building process.

**Fig 2:**
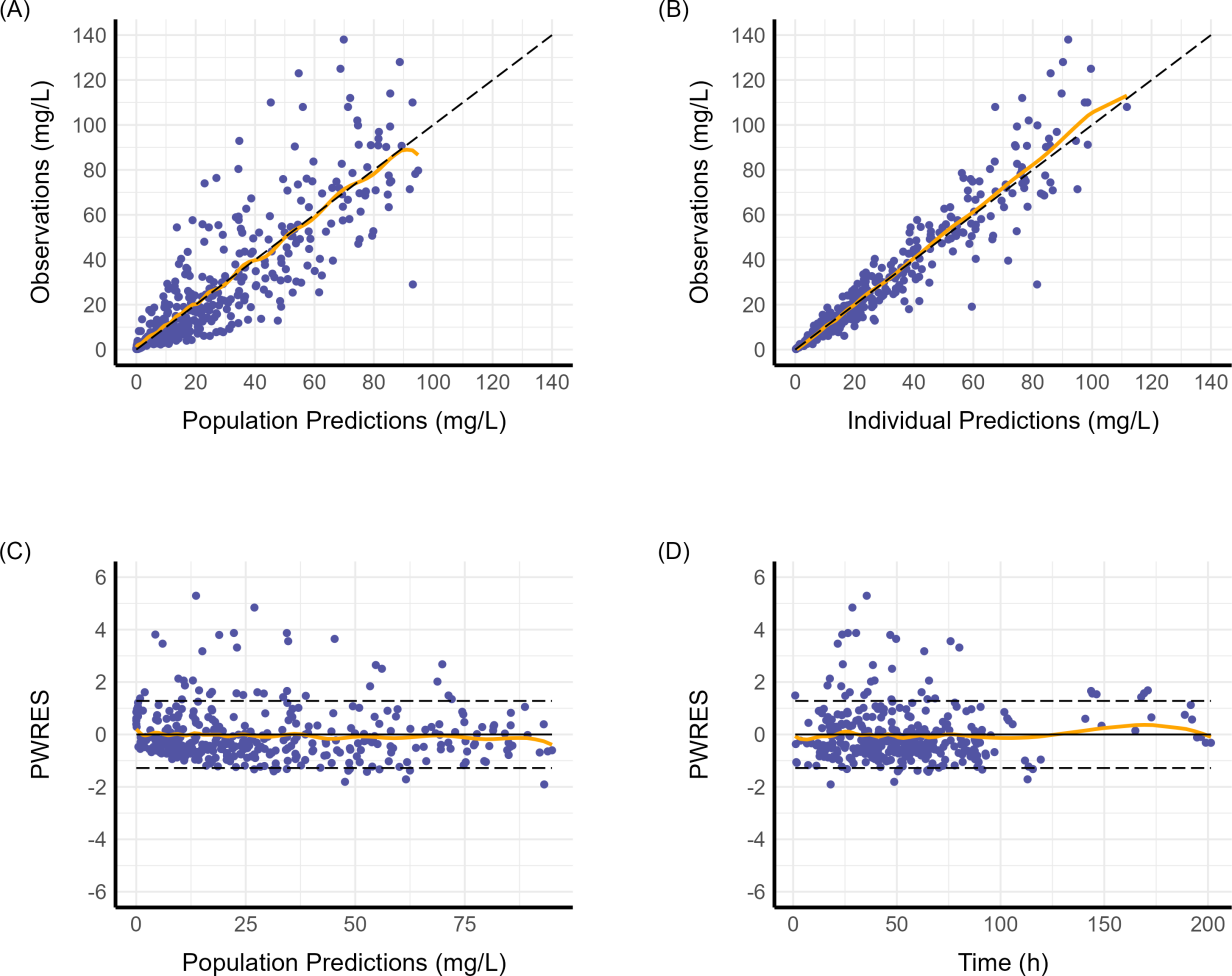
Diagnostic plots for the final population pharmacokinetic (PK) model. Population predictions (**A**) and Individual predictions (**B**) vs observations plots. Scatterplots of population-predicted concentrations (**C**) and time (**D**) vs population-weighted residuals. Yellow lines reflect loess best fit lines.

**Fig 3:**
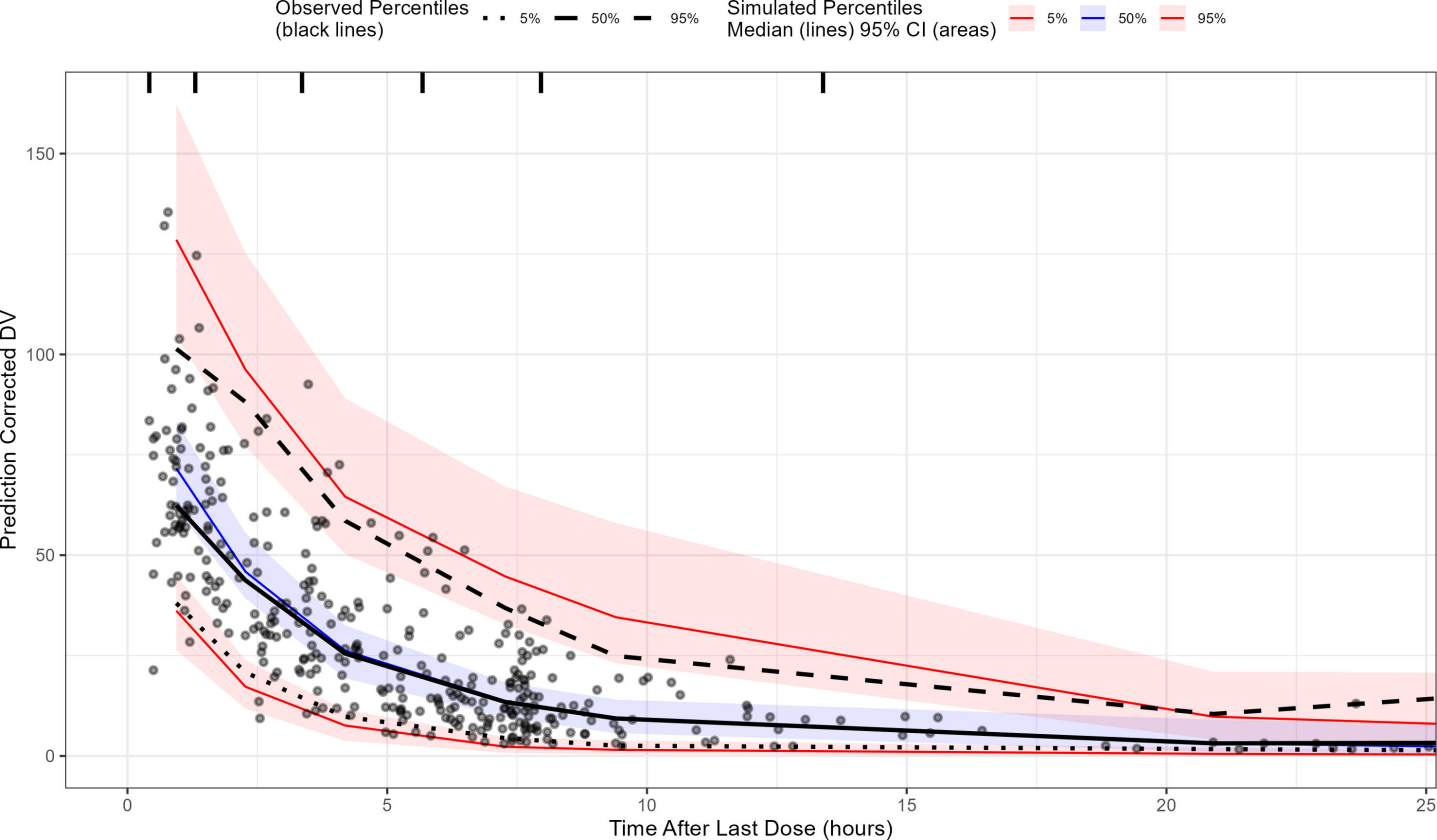
Visual predictive check of the final pharmacokinetic (PK) model. Observations are shown as block dots. The lines represent the median and 95% confidence intervals of the predicted (blue/red) and observed (black) data. The areas represent the 95% confidence intervals for the predicted percentiles.

Normalized to a 36-kg child, the final model parameter estimates for clearance were 5.14 L/h^0.75^ with 39% BSV, and the central volume was 20.53 L with 28% BSV (Table 2). Inter-compartmental clearance was estimated to be 0.64 L/h^0.75^, and V2 was estimated to be 4.76 L. The Empirical Bayes Estimates (i.e., most probable prediction for the individuals) for CL and V1 in the 4 patients who received ECMO were similar to the estimates in the overall population (5.42 L/h and 21.2 L, respectively). Goodness of fit plots (Fig. 2) and a visual predictive check (Fig. 3) demonstrated that the model fit the data well. Although the model under-predicted some of the observed concentrations, the overall prediction was reasonable as demonstrated by the LOESS fit line, which approximated the line of unity on both the observed vs population predicted and weighted residual plots. Based on the whole blood:plasma ratio, the estimated plasma CL and V1 would be 2.97 L/h and 11.5 L, respectively (Table 3).

**TABLE 2:** Final VAMS population pharmacokinetic parameter estimates^*b*^^,^^*c*^

Parameter	Estimate	Standard error	RSE%	95% CI
CL (L/h)	5.14	0.33	6.55	4.53–5.84
V1 (L)	20.53	1.38	6.72	18.01–23.41
Q (L/h)	0.64	0.31	48.4	0.28–1.47
V2 (L)	4.76	1.5	31.5	2.67–8.48
θ_GFR_	0.47	0.043	9.23	0.38–0.55
θ_Age_	−0.20	0.058	28.5	−0.32–−0.09
BSV CL (CV%)^*a*^	0.38 (39.0%)	0.048	12.7	0.29–0.48
BSV V1 (CV%)^*a*^	0.28 (29.4%)	0.054	19.3	0.19–0.4
RV	0.25	0.011	4.21	0.23–0.27

^*a*^ BSV reported as SD and CV %.

^*b*^ 95% CI, 95% CI; BSV, between subject variability; CL, total body clearance; CV, coefficient of variation; GFR, glomerular filtration rate; Q, intercompartmental clearance; RSE, relative standard error; RV, residual variability; V1, central volume; V2, peripheral volume.

^*c*^ Equations for PK parameters: $CLi\;=\;CL\;*\;{\left(\frac{WT}{36}\right)}^{0.75}*\;{\left(\frac{GFR}{100}\right)}^{\theta_{GFR}}*\;\eta CL$$V1i\;=\;V1\;*\left(\frac{WT}{36}\right)*\;{\left(\frac{Age}{10}\right)}^{\theta_{Age}}*\;\eta V1$$Qi=Q*{\left(\frac{WT}{36}\right)}^{0.75}$$V2i\;=\;V2\;*\;\left(\frac{WT}{36}\right)$.

**TABLE 3:** Estimated plasma PK parameters using adjusted concentrations based on whole blood VAMS:plasma ratio^*a*^^,^^*b*^^,^^*c*^

Parameter	Estimate	SE	RSE%	95% CI	Equation
CL (L/h)	2.97	0.19	6.48	2.62–3.37	$CLi=CL*{\left(\frac{WT}{36}\right)}^{0.75}*{\left(\frac{GFR}{100}\right)}^{\theta_{GFR}}*\eta CL$
V1 (L)	11.51	0.93	8.12	9.83–13.49	$V1i=V1*\left(\frac{WT}{36}\right)*{\left(\frac{Age}{10}\right)}^{\theta_{Age}}*\eta V1$
Q (L/h)	0.46	0.27	57.8	0.18–1.20	$Qi=Q*{\left(\frac{WT}{36}\right)}^{0.75}$
V2 (L)	3.21	0.89	27.9	1.91–5.38	$V2i=V2*\left(\frac{WT}{36}\right)$
θ_GFR_	0.49	0.13	26.0	0.24–0.74	
θ_Age_	−0.20	0.06	27.7	−0.32–−0.09	
BSV CL (CV%)	0.37 (38.7%)	0.052	13.9	0.29–0.49	
BSV V1 (CV%)	0.29 (29.5%)	0.065	22.4	0.19–0.44	

^*a*^ BSV reported as SD and CV %.

^*b*^ A whole blood VAMS:plasma ratio of 0.58:1 used to derive estimated plasma PK parameters.

^*c*^ 95% CI, 95% CI; BSV, between-subject variability; CL, total body clearance; CV, coefficient of variation; GFR, glomerular filtration rate; Q, intercompartmental clearance; RSE, relative standard error; RV, residual variability; V1, central volume; V2, peripheral volume.

### Simulation and target attainment during MODS

Of our 37 subjects, 32 started cefepime on/after MODS onset. VAMS PK profiles were simulated for the first 24 h of dosing for these subjects. Using a target of 100% *f*T > MIC, we found that 31 subjects (97%) maintained simulated concentrations above 2 mg/L (i.e., 100% *f*T > MIC of 2), 23 (72%) at MIC = 4 mg/L, 13 (41%) at MIC = 8 mg/L, and 3 (9%) at MIC = 16 mg/L.

## DISCUSSION

We developed a population PK model for cefepime in pediatric patients with MODS utilizing VAMS. Our final model was a two-compartment, allometrically scaled model with U25 eGFR and age as covariates on CL and V1, respectively. While the number of compartments varies across studies (20–22), most models incorporate body size and renal function as a covariate for cefepime clearance. The inclusion of age as a covariate is less common but has been utilized in neonatal and infant populations (21, 22). Since our study population included subjects as young as a month old, our findings align.

It is noteworthy that our model is the first developed using whole blood VAMS as opposed to plasma samples. Differences in the measured concentrations between whole blood VAMS and plasma contributed to apparent differences in our final model parameter estimates compared to other reported plasma-based values. However, this was not unexpected as we found that the concentrations of cefepime in VAMS were 53%–57% of those in plasma in our previous method development study (9). Since a different laboratory was used for drug quantification for the current study, we confirmed those experiments and found a similar ratio (0.58), which explains the higher estimates of CL and V1 in VAMS versus plasma. Our study relied solely on VAMS for sample collection, which enabled robust PK sampling that would not have been possible with larger volume plasma samples. Thus, because of the benefits of this new sampling modality, our report on PK and future planned outcomes analyses focuses on VAMS-derived concentrations. Knowledge of the relationship between VAMS and plasma concentrations (and PK) will facilitate incorporation of both VAMS and traditional blood sampling approaches into future studies and clinical care.

Even with a focus on a population of children with MODS, the mean adjusted plasma parameter estimates of our model were similar to other non-VAMS published cefepime PK models in critically ill children. A one-compartment model by De Cacqueray et al. (with allometric scaling centered on a weight of 9 kg and eGFR [median 153 mL/min/1.73 m^2^] as a covariate on CL) reported a CL of 1.21 L/h (20). Based on our plasma model, a 9-kg child with an eGFR of 153 mL/min/1.73 m^2^ would have a mean estimated CL of 1.05 L/h. In a more recent study of critically ill children and young adults, Morales Junior et al. reported a two-compartment model with a median CL of 6.41 L/h/70kg^0.75^ and V1 of 15 L/70kg (23). Scaled to 70 kg and using an eGFR of 147 mL/min/1.73 m^2^ (median from that study), the expected plasma CL and V1 using our model would be 5.91 L/h and 22.4 L, respectively. Thus, despite our study focusing on critically ill children with MODS, cefepime PK was largely similar to previous pediatric reports. We hypothesize that this is because the underlying cause of MODS in our study was diverse (e.g., sepsis, trauma, surgery), and we obtained PK samples at varying times in relation to MODS onset. Although the majority of children in our study (95%) had active MODS at the time of PK sampling, antibiotic PK may be most dynamic during the period when organ dysfunction first develops (i.e., at MODS onset). Because our study enrolled children following confirmation of MODS, our findings are most reflective of children with established multiple organ dysfunction rather than those whose organ function is changing.

MODS may influence cefepime PK in important but heterogeneous ways depending on the cause. While we attempted to evaluate covariates that could describe additional variability in our model (severity of illness scores, fluid balance, ECMO, etc.), these factors did not significantly improve model fit in our study. Consistent with other studies of renally eliminated medications in children, estimates of renal function (i.e., creatinine clearance) are the most influential covariate after allometric scaling. A larger study may be needed to fully elucidate how varying causes of organ dysfunction influence antibiotic PK, while a longitudinal assessment can help describe how antibiotic PK changes surrounding MODS onset and resolution. Additionally, although immunoparalysis was not identified as a significant covariate on cefepime PK in this study, this phenotype may significantly influence antibiotic pharmacodynamics, which will be evaluated in future studies.

Most subjects with MODS in our study (59%) failed to maintain free VAMS cefepime concentrations above 8 mg/L throughout the first 24 h of treatment. Despite having organ dysfunction, target failure in these children appears to place them at risk for treatment failure for resistant pathogens that are still considered dose-dependent susceptible (24). Future AMPLE studies will evaluate how clinical and organ function outcomes are impacted by antibiotic concentrations attained during MODS. Although out of the scope of the current study, we aim to establish VAMS-specific therapeutic targets through these future investigations, which will be critical to the translation of VAMS into clinical practice.

Traditionally, therapeutic targets for antibiotics have been derived using plasma concentrations since plasma provides a practical matrix with which to link dose, measured concentration, and effect. Unbound plasma concentrations serve as a proxy for systemic exposure and infection site-specific concentrations and, when indexed to the bacterial MIC, correlate with efficacy. Since β-lactams like cefepime do not readily partition into red blood cells, most of the drug is retained in plasma, making it the preferred matrix for quantification. However, because the majority of the drug is retained in plasma, it follows that concentrations in plasma and whole blood are proportional, driven largely by the cellular volume of the blood (i.e., hematocrit). Our *in vitro* data support this, showing that plasma concentrations are approximately 66% higher than whole blood. Although clinical verification is needed, the use of an adjustment factor can allow translation of measured concentrations in VAMS to plasma or extrapolation of plasma-based therapeutic targets to VAMS. Ultimately, the optimal target for cefepime TDM in critically ill children is uncertain, and we hope that continued investigation in this area will demonstrate VAMS as a viable alternative to plasma and elucidate its potential utility for optimizing treatment and monitoring.

Ultimately, the population PK model developed here can serve as a Bayesian prior for evaluation of relationships between individual drug concentrations (i.e., target attainment) and outcomes in a future investigation. Understanding how and whether VAMS concentrations relate to clinical outcomes (e.g., microbiologic, organ function outcomes) is a critical future direction of our work, which we hope will inform the application of VAMS for routine TDM. While plasma-based MIC targets are most commonly used, derivation of VAMS-specific targets can provide clinical value considering the relative ease with which microsamples can be obtained (i.e., capillary stick). The benefits of VAMS include (i) very low and precise volumes that enable accurate drug measurements, (ii) sample stability that can facilitate reference laboratory analyses, and (iii) collection of blood via multiple routes (e.g., venipuncture, capillary stick, or existing catheter). Conversion of VAMS concentrations to plasma for the purposes of determining target attainment will eventually be an important step to compare outcomes data to the wealth of historical data.

There are important limitations to our study. As mentioned above, sampling occurred over three consecutive days, but the sampling schedule was not aligned with MODS onset. Measurement of cefepime at different points in MODS progression had the potential to introduce additional variability into our current analysis. However, we evaluated day of MODS as a covariate on CL and V1 but did not find it to be a significant factor for either parameter (Table S3). Additionally, our study was not designed to capture data before MODS onset. While this information was not critical for understanding cefepime PK in MODS in our study, additional studies are needed to understand how MODS onset and progression imparts changes on cefepime PK over time. Finally, the VAMS:plasma ratio used in our study was based on a series of *in vitro* experiments. Although confirmed across multiple laboratories, clinical validation is needed to evaluate the accuracy of this conversion ratio in practice.

In conclusion, we have developed the first population PK model for cefepime in critically ill children with MODS using VAMS. Our model parameter estimates appeared to differ slightly from previous reports, but this is likely because whole blood (VAMS) has approximately twice the volume of plasma. When corrections were made based upon our experimental data, plasma PK was similar to previous reports, but importantly, we have now defined the PK for cefepime using VAMS in children with MODS. Additional studies are needed to understand the relationships between cefepime concentrations, target attainment, and clinical outcomes when using VAMS.

## Data Availability

Data are available through NICHD’s Data and Specimen Hub (DASH) at the following link: https://dash.nichd.nih.gov/study/103755. This page provides study information and instructions for accessing the data set. Note that at the time of initial manuscript acceptance, NICHD is/was in the process of migrating DASH to a new platform. A link to the new data repository will be provided when available.

## SUPPLEMENTAL MATERIAL

Supplemental material aac.01736-25-s0001.docx Fig. S1; Tables S1 to S4.

ASM does not own the copyrights to Supplemental Material that may be linked to, or accessed through, an article. The authors have granted ASM a non-exclusive, world-wide license to publish the Supplemental Material files. Please contact the corresponding author directly for reuse.
