# Supplementary material for: Cefepime pharmacokinetics in critically ill children with multiple organ dysfunction syndrome using volumetric absorptive microsampling
Source: Antimicrob Agents Chemother. 2026 Jun 4;70(7):e01736-25. doi: 10.1128/aac.01736-25 (PMC13321836; doi:10.1128/aac.01736-25)
Supplement: Supplemental material — Fig. S1; Tables S1 to S4. [file aac.01736-25-s0001.docx]

**Figure S1. Observed concentration versus time after dose (in hours).**

**
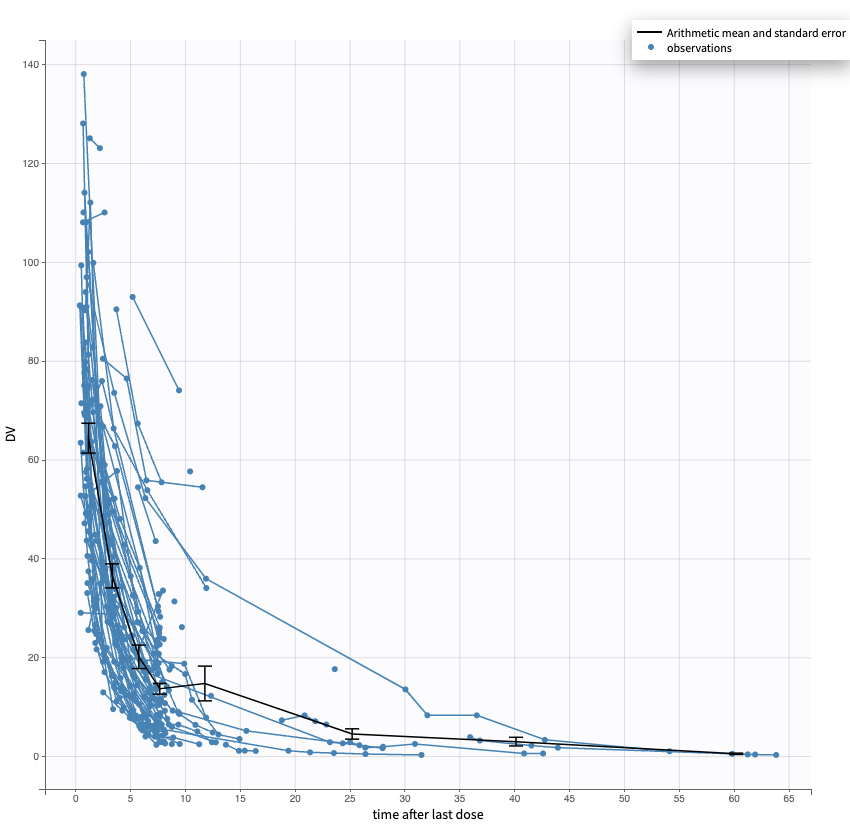
**

Blue dots represent observed VAMS concentrations and blue lines connect observations from a single individual. The black shows the arithmetic mean of all observations with standard errors. Y-axis displays the measured concentration (in mg/L), or dependent variable (DV), and x-axis is time that that the observation was collected after the preceding dose (in hours).

**Table S1: Inclusion and Exclusion Criteria**

**Inclusion Criteria**

1. Pediatric ICU (PICU) or Cardiothoracic ICU/Cardiac ICU (CTICU/CICU) Admission
2. At least 40 weeks post-conceptual age and < 18 years old at time of consent (subject becomes ineligible for new enrollment on 18^th^ birthday)
3. Patients who have received myeloablative therapy (chemotherapy or radiation) in the past month (30 days), must have a white blood cell (WBC) count > 1,000 cells/mm3 in the 24 hours prior to the time of consent
4. Patients receiving or planning to receive 1 or more of the qualifying intravenous (IV) antibiotics:
   1. Vancomycin
   2. Piperacillin/Tazobactam
   3. Cefepime
   4. Meropenem
   5. Ceftriaxone
   6. Ampicillin
   7. Ampicillin/Sulbactam
5. Acute multiple organ dysfunction syndrome (MODS), defined as onset of ≥2 organ dysfunctions within the past 3 calendar days (see below)

**Exclusion Criteria**

1. Presence of limitation of care orders at the time of screening
2. Clinical suspicion of (or ongoing evaluation for) brain death
3. Previous enrollment in PediAtric ReseArch of Drugs, Immunoparalysis, and Genetics during MODS (PARADIGM) or PARADIGM-Assessment of MODS and PersonaLized Exposures of Antibiotics (AMPLE) study

**Organ Dysfunction Diagnostic Criteria**

**a) Respiratory Dysfunction**

- Respiratory rate > 90 breaths per min (age < 1yr) or > 70 breaths per min (age ≥ 1yr) **OR**
- PaO2 < 40 mmHg (5.3kPa) in absence of cyanotic congenital heart disease **OR**
- PaCO2 ≥ 65 mmHg (8.7kPa) **OR**
- PaO2/FiO2 < 200 in absence of cyanotic congenital heart disease (where P/F ratio is based on simultaneous measurements) **OR**
- Mechanical ventilation
  - Must be for > 24hrs post-operative if intubated just for surgery/procedure
  - Includes new HFNC ≥ 6L/min, non-invasive ventilation (e.g. CPAP, BiPAP, SiPAP), and veno-venous (VV) or veno-arterial (VA) extracorporeal membrane oxygenation (ECMO)
  - Includes patients with tracheostomy who do NOT require mechanical ventilation at baseline
  - For subjects with non-invasive ventilation at baseline (e.g. CPAP for obstructive sleep apnea), intubation would qualify as Respiratory Dysfunction

**b) Cardiovascular Dysfunction**

- Systolic blood pressure < 40 mmHg (age < 1yr) or < 50 mmHg (age ≥ 1yr) **OR**
- Heart rate < 50 or > 220 (age < 1yr), < 40 or > 200 (age ≥1yr) **OR**
- Cardiac arrest **OR**
- pH < 7.2 with normal PaCO2 **OR**
- Continuous vasoactive drug infusion for hemodynamic support (excluding dopamine infusion ≤5ug/kg/min) **OR**
- VA ECMO **OR**
- Ventricular assist device

**c) Hematologic Dysfunction**

- Hemoglobin < 5g/dL (50g/L) **OR**
- White blood cell count < 3,000/mm^3^ (3x10^9/L) **OR**
- Platelet count < 20,000/mm^3^ (20x10^9) **OR**
- PT > 20 seconds **OR** aPTT > 60 seconds (in absence of anticoagulation therapy)

**d) Gastrointestinal Dysfunction**

- Gastrointestinal bleeding **AND** one of the following believed to be the result of gastroduodenal bleeding by the treating physician
  - Drop in hemoglobin ≥ 2g/dL (20g/L) over 24 hours **OR**
  - Blood transfusion **OR**
  - Hypotension with blood pressure < 5th percentile of age **OR**
  - Gastric or duodenal surgery

**e) Renal Dysfunction**

- Serum BUN ≥ 100mg/dL (36 mmol/L) **OR**
- Serum creatinine ≥ 2 mg/dL (177 μmol/L) without pre-existing renal disease **OR**
- Dialysis and/or hemofiltration

**f) Neurological Dysfunction**

- Fixed, dilated pupils **OR**
- Glasgow coma score (GCS) < 5 in the absence of neuromuscular blocking drugs

**g) Hepatic Dysfunction**

- Total bilirubin > 3mg/dL (60 μmol/L)

**Table S2. Cefepime volumetric absorptive microsampling (VAMS) whole blood-to-plasma ratio.** *In vitro* experiments were performed over a range of concentrations. For each quality control (QC) run, 6 whole blood samples were spiked with known quantity of cefepime to create varying concentrations of cefepime in whole blood. These samples were then loaded to VAMS devices in duplicate and the remaining whole blood was spun to create plasma aliquots (x2). Concentrations were simultaneously measured in whole blood and plasma, and the ratio was derived for each of the paired samples.

| QC Run^a^ | Known Concentration (mg/L) | VAMS average concentration (mg/L) | Plasma average concentration (mg/L) | Average Ratio VAMS whole blood:plasma^b^ |
| --- | --- | --- | --- | --- |
| 1 | 0.2 | 0.22 | 0.36 | 0.61 |
| 2 | 2 | 2.17 | 3.74 | 0.58 |
| 3 | 40 | 46.1 | 74.5 | 0.62 |
| 4 | 80 | 84.3 | 142.3 | 0.59 |
| 5 | 150 | 139.6 | 268.8 | 0.52 |
|  | | | **Overall ratio** | **0.58** |
| ^a^ Each QC run reflects 6 samples run in duplicate | | | | |
| ^b^ The ratios reported reflect the average of the 6 paired samples per QC run rather than the ratio of the average concentrations. The overall ratio is the average of all paired runs (n=30), rather than the average of the 5 average ratios. | | | | |

| Table S3. Daily characteristics of study population.^a^ | | | | | |
| --- | --- | --- | --- | --- | --- |
| MODS Day | SCr (mg/dL), median (range) | U25 eGFR (mL/min/1.73 m^2^), median (range) | Mechanical ventilation, n (%) | Vasopressor use, n (%) | PELOD-2 score, median (range) |
| 0 (n= 3) | 0.45 (0.15 - 1.22) | 83 (48 - 278) | 3 (100.0%) | 3 (100.0%) | 5 (5 - 8) |
| 1 (n=18) | 0.61 (0.16 - 1.62) | 80 (19 - 259) | 17 (94.4%) | 15 (83.3%) | 7 (0 - 21) |
| 2 (n=24) | 0.48 (0.16 - 2.77) | 104 (21 - 244) | 24 (100.0%) | 22 (91.7%) | 7 (0 - 13) |
| 3 (n=18) | 0.48 (0.24 - 2.96) | 97 (19 - 163) | 17 (94.4%) | 13 (72.2%) | 6 (1 - 11) |
| 4 (n=9) | 0.53 (0.30 - 2.61) | 98 (22 - 152) | 8 (88.9%) | 3 (33.3%) | 5 (1 - 15) |
| 5 (n=1) | 0.36 (0.36 - 0.36) | 101 (101 - 101) | 0 (0.0%) | 0 (0.0%) | 2 (2 - 2) |
| 6 (n=1) | 0.31 (0.31 - 0.31) | 163 (163 - 163) | 1 (100.0%) | 0 (0.0%) | 7 (7 - 7) |
| 7 (n=2) | 0.27 (0.23 - 0.31) | 131 (99 - 163) | 1 (50.0%) | 0 (0.0%) | 4 (0 - 7) |
| 8 (n=2) | 0.90 (0.30 - 0.95) | 78 (74 - 169) | 2 (100.0%) | 0 (0.0%) | 4 (3 - 5) |
| 9 (n=3) | 0.48 (0.37 - 0.75) | 123 (82 - 164) | 3 (100.0%) | 0 (0.0%) | 4 (3 - 9) |

^a^ Number of individuals reported for each MODS Day reflects the number of patients who both received cefepime and underwent PK sampling that day.

| **Table S4: Phase 1 Forward Addition**  Of the covariates tested, U25 eGFR on CL was found the have the highest significant reduction in OFV and met all other selection criteria. U25 on CL was retained in the model going forward. | | | | | | |
| --- | --- | --- | --- | --- | --- | --- |
| Model Name | OFV | ΔOFV | AIC | BICc | ΔBICc | Eigen Value |
| Base Model | 2454.91 | --- | 2468.91 | 2491.79 | --- | 16.15 |
| + U25 GFR on CL | 2405.88 | -49.03 | 2421.88 | 2448.69 | -43.1 | 2.62 |
| + eGFR on CL | 2407.17 | -47.74 | 2423.17 | 2449.99 | -41.8 | 17.94 |
| + Day on CL | 2435.98 | -18.93 | 2451.98 | 2478.8 | -12.99 | 19.8 |
| + Day on V | 2442.99 | -11.92 | 2458.99 | 2485.8 | -5.99 | 17.23 |
| + PELOD-2 on CL | 2437.21 | -17.7 | 2453.21 | 2480.03 | -11.76 | 11.77 |
| + Proulx on CL | 2445.89 | -9.02 | 2461.89 | 2488.71 | -3.08 | 12.39 |
| + Age on V | 2447.58 | -7.33 | 2463.58 | 2488.08 | -3.71 | 37.54 |
| + Proulxon V | 2447.74 | -7.17 | 2463.74 | 2490.56 | -1.23 | 29.07 |
| + Vent on CL | 2447.9 | -7.01 | 2463.9 | 2490.72 | -1.07 | 16.98 |
| + PELOD-2 on V | 2448.51 | -6.4 | 2464.51 | 2491.32 | -0.47 | 2.81 |
| + Vaso on V | 2448.52 | -6.39 | 2464.52 | 2491.34 | -0.45 | 7.57 |
| + PRISM on V | 2450.23 | -4.68 | 2466.23 | 2493.05 | 1.26 | 4.5 |
| + Gender on V | 2450.99 | -3.92 | 2466.99 | 2491.48 | -0.31 | 55.72 |
| + ECMO on V | 2451.1 | -3.81 | 2467.1 | 2493.92 | 2.13 | 10.79 |
| + Vaso on CL | 2451.35 | -3.56 | 2467.35 | 2494.16 | 2.37 | 14.53 |
| + PRISM on CL | 2451.4 | -3.51 | 2467.4 | 2494.22 | 2.43 | 22.82 |
| + Immune on CL | 2451.66 | -3.25 | 2467.66 | 2492.15 | 0.36 | 14.81 |
| + Gender on CL | 2452.2 | -2.71 | 2468.2 | 2492.69 | 0.9 | 27.58 |
| + Vent on V | 2452.23 | -2.68 | 2468.23 | 2495.05 | 3.26 | 8.22 |
| + TNF on CL | 2452.65 | -2.26 | 2468.65 | 2495.47 | 3.68 | 20.72 |
| + Age on CL | 2452.7 | -2.21 | 2468.7 | 2493.2 | 1.41 | 20.24 |
| + Fluids on V | 2452.9 | -2.01 | 2468.9 | 2495.72 | 3.93 | 5.07 |
| + Fluids on CL | 2453.94 | -0.97 | 2469.94 | 2496.76 | 4.97 | 11.15 |
| + Immune on V | 2454.06 | -0.85 | 2470.06 | 2494.55 | 2.76 | 24.28 |
| + TNF on V | 2454.2 | -0.71 | 2470.2 | 2497.01 | 5.22 | 15.01 |
| + ECMO on CL | 2455.11 | 0.2 | 2471.11 | 2497.93 | 6.14 | 9.16 |
| eGFR = Bedside Schwartz eGFR  U25 = Chronic Kidney Disease in Children Under 25 eGFR equation  Vent = ventilator use (yes/no)  Vaso = vasopressor use (yes/no)  ECMO = ECMO use (yes/no)  Immune = immunoparalysis based on TNF-α response (yes/no)  TNF = TNF-alpha response  Day = Days since MODS onset  Fluids = Daily net fluids (+/-)  Abbreviations: PRISM, Pediatric Risk of Mortality score; PELOD-2 = Pediatric Logistic Organ Dysfunction-2 score; OFV, objective function value; AIC, Akaike Information Criterion; BICc, corrected Bayesian Information Criterion | | | | | | |

| **Table S4 (continued): Phase 2 Forward Addition**  Of the covariates tested, Age on V was found the have the highest significant reduction in OFV and met all other selection criteria. Age on V was retained in the model going forward. | | | | | | | |
| --- | --- | --- | --- | --- | --- | --- | --- |
| Model Name | OFV | ΔOFV | AIC | BICc | ΔBICc | Eigen Value | |
| Base + U25 | 2405.88 | --- | 2421.88 | 2448.69 | --- | 2.62 | |
| + Age on V | 2395.53 | -10.35 | 2413.53 | 2441.96 | -6.73 | 44.93 | |
| + Fluids on V | 2398.49 | -7.39 | 2416.49 | 2447.24 | -1.45 | 69.21 | |
| + Fluids on CL | 2398.71 | -7.17 | 2416.71 | 2447.45 | -1.24 | 9.35 | |
| + Proulxon V | 2399.58 | -6.3 | 2417.58 | 2448.33 | -0.36 | 13.77 | |
| + PELOD-2 on V | 2400.29 | -5.59 | 2418.29 | 2449.04 | 0.35 | 4.22 | |
| + Vent on V | 2400.41 | -5.47 | 2418.41 | 2449.16 | 0.47 | 12.78 | |
| + PELOD-2 on CL | 2400.65 | -5.23 | 2418.65 | 2449.4 | 0.71 | 77.56 | |
| + Day on CL | 2400.95 | -4.93 | 2418.95 | 2449.7 | 1.01 | 53.54 | |
| + Proulxon CL | 2401.19 | -4.69 | 2419.19 | 2449.94 | 1.25 | 18.78 | |
| + PRISM on CL | 2401.56 | -4.32 | 2419.56 | 2450.3 | 1.61 | 23.46 | |
| + Day on V | 2401.69 | -4.19 | 2419.69 | 2450.44 | 1.75 | 6.01 | |
| + Vent on CL | 2402.78 | -3.1 | 2420.78 | 2451.53 | 2.84 | 10.92 | |
| + Immune on CL | 2402.95 | -2.93 | 2420.95 | 2451.7 | 3.01 | 3.81 | |
| + Gender on V | 2403.03 | -2.85 | 2421.03 | 2449.45 | 0.76 | 20.21 | |
| + Gender on CL | 2403.54 | -2.34 | 2421.54 | 2449.96 | 1.27 | 81.8 | |
| + Vaso on V | 2404.58 | -1.3 | 2422.58 | 2453.32 | 4.63 | 19.04 | |
| + ECMO on CL | 2405.23 | -0.65 | 2423.23 | 2453.98 | 5.29 | 174.51 | |
| + Vaso on CL | 2405.58 | -0.3 | 2423.58 | 2454.32 | 5.63 | 10.68 | |
| + TNF on V | 2405.78 | -0.1 | 2423.78 | 2454.53 | 5.84 | 25.57 | |
| + AGE on CL | 2405.89 | 0.01 | 2423.89 | 2452.32 | 3.63 | 11.31 | |
| + TNF on CL | 2406.07 | 0.19 | 2424.07 | 2454.81 | 6.12 | 38.85 | |
| + ECMO on V | 2406.97 | 1.09 | 2424.97 | 2455.72 | 7.03 | 82.88 | |
| + PRISM on V | 2411.06 | 5.18 | 2429.06 | 2459.81 | 11.12 | 682.44 | |
| + Immune on V | 2413.98 | 8.1 | 2431.98 | 2462.73 | 14.04 | 18.45 | |
| eGFR = Bedside Schwartz eGFR  U25 = Chronic Kidney Disease in Children Under 25 GFR  Vent = ventilator use (yes/no)  Vaso = vasopressor use (yes/no)  ECMO = ECMO use (yes/no)  Immune = immunoparalysis based on TNF-α response (yes/no)  TNF = TNF-alpha response  Day = Days since MODS onset  Fluids = Daily net fluids (+/-)  Abbreviations: PRISM, Pediatric Risk of Mortality score; PELOD-2 = Pediatric Logistic Organ Dysfunction-2 score; OFV, objective function value; AIC, Akaike Information Criterion; BICc, corrected Bayesian Information Criterion | | | | | | | |

| **Table S4 (continued): Phase 3 Forward Addition**  While Proulx on V, PELOD on V and CL, and Fluids on V and CL all met the OFV and BICc criteria, they were not considered for the final model. PELOD on CL and Proulx on V failed to improve BSV on their respective parameters. PELOD on V did reduce BSV on V, but its effect size was small and the addition worsened goodness of fit plots. Fluid status on CL and V reduced BSV in both cases, but the models were unstable and failed to estimate some values. The full model includes U25 GFR on CL and Age on V. | | | | | | |
| --- | --- | --- | --- | --- | --- | --- |
| Model Name | OFV | ΔOFV | AIC | BICc | ΔBICc | Eigen Value |
| Base + U25 + Age | 2395.53 | --- | 2413.53 | 2441.96 | --- | 44.93 |
| + Fluids on V | 2387.14 | -8.39 | 2407.14 | 2439.50 | -2.46 | *NAN* |
| + Proulxon V | 2387.7 | -7.83 | 2407.7 | 2440.06 | -1.9 | 2.43 |
| + PELOD-2 on V | 2389.14 | -6.39 | 2409.14 | 2441.5 | -0.46 | 19.8 |
| + PELOD-2 on CL | 2389.24 | -6.29 | 2409.24 | 2441.6 | -0.36 | 16.94 |
| + Fluids on CL | 2389.32 | -6.21 | 2409.32 | 2441.68 | -0.28 | *NAN* |
| + Immune on CL | 2389.74 | -5.79 | 2409.74 | 2442.1 | 0.14 | 28.56 |
| + Vent on CL | 2389.75 | -5.78 | 2409.75 | 2442.11 | 0.15 | 11.1 |
| + Day on CL | 2390.19 | -5.34 | 2410.19 | 2442.54 | 0.58 | 40.28 |
| + Proulxon CL | 2390.36 | -5.17 | 2410.36 | 2442.72 | 0.76 | 2.25 |
| + Day on V | 2391.35 | -4.18 | 2411.35 | 2443.71 | 1.75 | 80.62 |
| + Gender on V | 2391.8 | -3.73 | 2411.8 | 2441.83 | -0.13 | 78.33 |
| + Vaso on V | 2392.09 | -3.44 | 2412.09 | 2444.45 | 2.49 | 88.07 |
| + PRISM on CL | 2392.45 | -3.08 | 2412.45 | 2444.81 | 2.85 | 6.05 |
| + Gender on CL | 2393.26 | -2.27 | 2413.26 | 2443.3 | 1.34 | 18.84 |
| + Vent on V | 2393.48 | -2.05 | 2413.48 | 2445.84 | 3.88 | 32.61 |
| + AGE on CL | 2394.53 | -1 | 2414.53 | 2444.57 | 2.61 | 33.29 |
| + ECMO on CL | 2394.66 | -0.87 | 2414.66 | 2447.02 | 5.06 | 34.09 |
| + Vaso on CL | 2395.04 | -0.49 | 2415.04 | 2447.4 | 5.44 | 2.44 |
| + Immune on V | 2395.23 | -0.3 | 2415.23 | 2447.59 | 5.63 | 24.09 |
| + ECMO on V | 2395.24 | -0.29 | 2415.24 | 2447.6 | 5.64 | 1980.13 |
| + PRISM on V | 2406.49 | 10.96 | 2426.49 | 2458.85 | 16.89 | 130.46 |
| + TNF on CL | 2413.75 | 18.22 | 2433.75 | 2466.11 | 24.15 | 7.95 |
| eGFR = Bedside Schwartz eGFR  U25 = Chronic Kidney Disease in Children Under 25 GFR  Vent = ventilator use (yes/no)  Vaso = vasopressor use (yes/no)  ECMO = ECMO use (yes/no)  Immune = immunoparalysis based on TNF-α response (yes/no)  TNF = TNF-alpha response  Day = Days since MODS onset  Fluids = Daily net fluids (+/-)  *NAN* = “Not a Number” output given by Monolix when value fails to estimate  Abbreviations: PRISM, Pediatric Risk of Mortality score; PELOD-2 = Pediatric Logistic Organ Dysfunction-2 score; OFV, objective function value; AIC, Akaike Information Criterion; BICc, corrected Bayesian Information Criterion | | | | | | |

| **Table S4 (continued): Phase 4 Backwards Elimination**  Removing each covariate significantly increased the OFV. Both will be retained in the final model. | | | | | | |
| --- | --- | --- | --- | --- | --- | --- |
| Model Name | OFV | ΔOFV | AIC | BICc | ΔBICc | Eigen Value |
| Complete Model | 2395.53 | --- | 2413.53 | 2441.96 | --- | 44.93 |
| - Age | 2405.88 | 10.35 | 2421.88 | 2448.69 | 6.73 | 2.62 |
| - U25 GFR | 2447.58 | 52.05 | 2463.58 | 2488.08 | 46.12 | 37.54 |
